# Exploration of tumor size measurement methods in preoperative breast cancer assessment using whole-body silicon photomultiplier PET: feasibility and first results

**DOI:** 10.1007/s11604-024-01533-3

**Published:** 2024-02-12

**Authors:** Hiroyuki Kuroda, Takeshi Yoshizako, Nobuhiro Yada, Tomomi Kamimura, Nobuko Yamamoto, Mitsunari Maruyama, Rika Yoshida, Mizuki Fukuda, Yuko Kataoka, Masayuki Itakura, Yasushi Kaji

**Affiliations:** 1 Department of Radiology, Faculty of Medicine, Shimane University, 89-1 Enya, 00693-8501, Izumo, Shimane Japan; 2 Department of Radiology, Shimane University Hospital, Izumo, Shimane Japan; 3 Pathology Division, Faculty of Medicine, Shimane University, Izumo, Shimane Japan; 4 Department of Digestive and General Surgery, Faculty of Medicine, Shimane University, Izumo, Shimane Japan

**Keywords:** Breast cancer, SiPM PET, Prone position, Whole-body PET, Tumor size

## Abstract

**Purpose:**

Whole-body silicon photomultiplier positron emission tomography (WB SiPM PET) could be used to diagnose breast cancer spread before lumpectomy. We aimed to investigate the method of measuring the tumor size by WB SiPM PET as a basis for diagnosing breast cancer spread in the breast.

**Materials and methods:**

We retrospectively reviewed 35 breast cancer lesions in 32 patients who underwent WB SiPM PET/CT in the prone position as preoperative breast cancer examinations from September 2020 to March 2022.

In all cases, a 20-mm spherical VOI was placed in the normal mammary gland to measure the mean standardised uptake value (SUVmean) and the standard deviation (SD) of ^18^F-fluorodeoxyglucose (FDG) uptake. We prepared four types of candidates (SUVmean + 2 SD, SUVmean + 3 SD, 1.5 SUVmean + 2 SD, 1.5 SUVmean + 3 SD) for thresholds for delineating tumor contours on PET images. On the semiautomatic viewer soft, the maximum tumor sizes were measured at each of the four thresholds and compared with the pathological tumor sizes, including the extensive intraductal component (EIC).

**Results:**

The lesion detection sensitivity was 97% for WB SiPM PET. PET detected 34 lesions, excluding 4-mm ductal carcinomas in situ (DCIS). PET measurements at the '1.5 SUVmean + 2 SD' threshold demonstrated values closest to the pathological tumor sizes, including EIC. Moreover, '1.5 SUVmean + 2 SD' had the highest concordance (63%).

**Conclusions:**

The study demonstrated that among various PET thresholds, the '1.5 SUVmean + 2 SD' threshold exhibited the best performance. However, even with this threshold, the concordance rate was limited to only 63%.

## Introduction

The development of dedicated high-resolution breast positron emission tomography (breast PET) has made it possible to diagnose the spread of breast cancer within the breast, which had been impossible with conventional PET/CT due to its low resolution [1, 2]. Breast PET requires experts to overcome high hurdles to installing it in hospitals, such as the need for a dedicated imaging room. On the other hand, whole-body silicon photomultiplier PET (WB SiPM PET) also has a higher spatial resolution than the conventional photomultiplier tube detector, and it is expected that these devices will become widespread in many hub hospitals for cancer treatment not long from now.

In breast-conserving surgery, the extent of resection is determined by the size of the tumor, the number of tumor foci, and the locations of these foci [3]. Since the tumor size looks different depending on the threshold employed in nuclear medicine examination, the establishment of a measurement method that reflects the pathological tumor size is the basis for the diagnosis of the spread of cancer in the breast. However, there is no established method for measuring breast cancer tumor size via ^18^F-fluorodeoxyglucose PET (FDG-PET).

This retrospective study aimed to investigate the method of measuring the tumor size by WB SiPM PET as a basis for diagnosing the spread of breast cancer in the breast by WB SiPM PET.

## Materials and methods

### Patients

Patients who were newly diagnosed with breast cancer from September 2020 to March 2022 and scheduled for breast surgery within 2 months at our hospital were the primary subject group. The patients had been initially diagnosed via mammography, breast ultrasonography, or palpation. The lesions were confirmed to be breast cancer through biopsy and histopathology. Among these patients, those who underwent FDG-PET/computed tomography (FDG-PET/CT) for initial staging were retrospectively enrolled. Patients were excluded if neoadjuvant chemotherapy was performed.

### FDG-PET/CT protocol

**Fig. 1:**
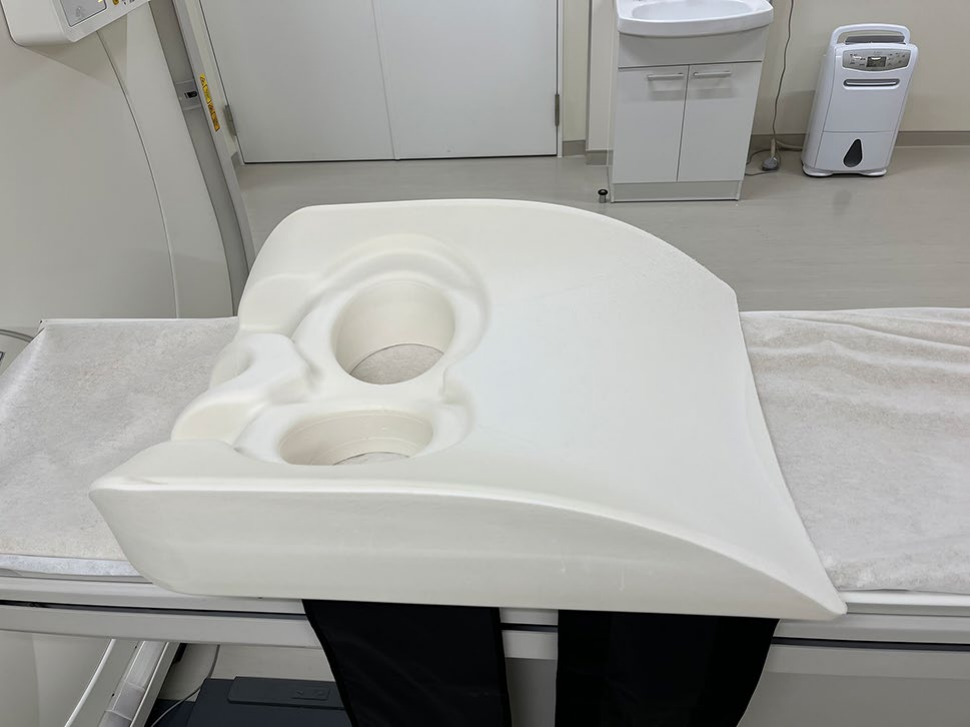
Prone positioner made of polyethylene foam

All patients were injected with FDG (3.7 MBq/kg) after fasting for at least six hours. Then, after resting for 60 min, the whole-body was imaged in the supine position. One hundred and twenty minutes after FDG injection, the breasts and regional lymph node areas were imaged by suspending the breast using a prone positioner (Philips Healthcare, OH, USA) (Fig. 1).

The PET data were acquired at 5 min/bed in the 3D list-mode using the SiPM-based TOF PET/CT system (Vereos PET/CT, multi-slice CT scanner, Philips Healthcare, OH, USA). The data were reconstructed using the 3D-list mode-based TOF OSEM algorithm (iterations 4, subsets 10) without post filtering. The voxel size was 2.00 × 2.00 × 2.00 mm^3^. Monte Carlo-based scatter simulation was applied for scatter correction and Casey averaging was applied for smoothed random estimation. The CT data were acquired for attenuation correction under the following conditions: tube voltage, 120 kV; absolute minimum tube-current time product, 30 mAs; iDose4, level 4.

### Histopathological examination

The samples for histopathological examination were prepared by making serial 5-mm slices of surgical specimens. Histological diagnosis was performed by a pathologist with at least 5 years of experience in breast histology. The tumor size, type and nuclear grade were documented from surgical pathology. The pathological tumor sizes were evaluated, including EIC. Correlations between the histological findings and the breast imaging findings were examined by the radiologists and pathologist in all cases.

### Image analysis

**Fig. 2:**
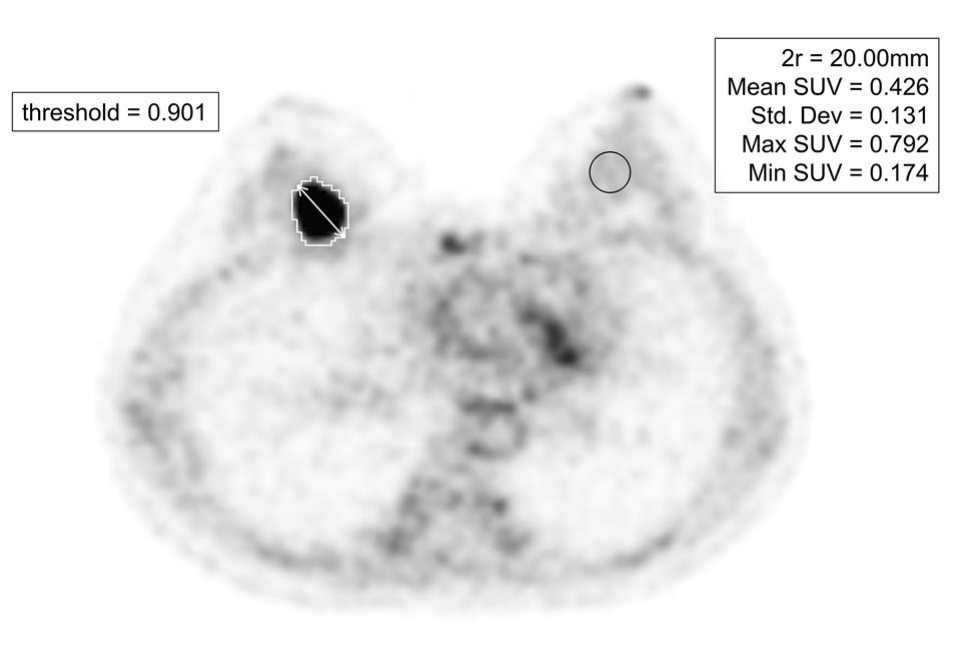
Example of semiautomatic delineation of tumor contours with patient-specific thresholds. The VOI to measure the uptake of background on left normal breast tissue (Black line). The auto-delineated tumor contour with its patient-specific threshold (White line). The tumor’s extent varies at four different thresholds. The '1.5 SUVmean + 2 SD' threshold (= 0.901) is presented as an example. Maximum diameter of the tumor (Double-headed arrow)

The maximal diameter of the lesions was measured using WB SiPM PET, and compared with the pathological tumor size, including EIC. A commercially available imaging viewer (XTREK, J-MAC system, Sapporo, Japan) was used for measurements on images of all PET scans. A spherical volume of interest (VOI) with a diameter of 20 mm was placed in the contralateral normal mammary gland to measure the background activity. We positioned the VOI in the center of the area with high FDG accumulation in the contralateral mammary gland, avoiding the area directly below the nipple where physiological accumulation is high. When breast cancer was present on both sides, the VOI was placed in a position distant from the lesion in either the left or right mammary gland. The mean standardised uptake value (SUVmean) and the standard deviation (SD) of the SUV in the spherical VOI are measured (to three decimal places). Four types of patient-specific threshold candidates were prepared according to the PERCIST method: SUVmean + 2 SD, SUVmean + 3 SD, 1.5 SUVmean + 2 SD, and 1.5 SUVmean + 3 SD [4, 5]. Tumors were semiautomatically delineated by patient-specific thresholds, maximum diameters were measured by two radiologists with 34 and 7 years of experience respectively, and the average values were calculated (Fig. 2). We measured the maximum diameter of the tumor as the largest value among the three sections: axial, coronal, and sagittal. The maximum tumor sizes were measured at each of the four thresholds on semiautomatic viewer soft and compared with the pathological tumor sizes, including EIC to determine the optimal patient-specific threshold.

### Statistical analysis

The Shapiro–Wilk test was used to determine whether the mean values were normally distributed. Differences between pathology and the four threshold groups were analysed by the Friedman test and Wilcoxon signed rank test with Bonferroni correction as a post hoc test after confirming a distribution pattern by using the Shapiro–Wilk test.

Next, the correlations between pathology and the four threshold groups were assessed by Spearman’s rank correlation analysis. The strengths of the correlation coefficients (r_s_) were interpreted as either no correlation (0.00–0.19) or weak (0.20–0.39), moderate (0.40–0.69), and strong (0.70–1.00) correlations.

All statistical analyses were performed using IBM SPSS version 22.0 for Windows (IBM Corp.: Armonk, NY), and a p-value of < 0.05 was considered statistically significant.

## Results

### Patient and Tumor characteristics

We retrospectively reviewed 35 breast cancer lesions in 32 patients who underwent WB SiPM (Table 1). The participants underwent curative surgery. Of these, partial mastectomies were performed for 20 lesions, and total mastectomies for 15 lesions. In all cases, the resection margins were negative. The ages of our participants (who were all women) ranged from 34 to 87 years, with a mean value of 60.4 years. One of the 32 patients had bilateral breast cancer. Daughter lesions were present in 2 out of 33 breasts, and the total number of index lesions was 35. The tumors under study consisted of four ductal carcinomas in situ (DCIS), two microinvasive carcinomas, 25 invasive ductal carcinomas (IDC) and four invasive lobular carcinomas (ILC). According to the intrinsic subtype classification, the tumors were distributed as follows: 20 were classified as Luminal A, 10 as Luminal B, one as Her 2 and 4 as Triple-negative [6]. Pathological tumor invasion size ranged from 0 (in DCIS) to 42 mm, with a median value of 13.5 mm. The pathological tumor size, including EIC, ranged from 4 to 110 mm, with a median value of 23.3 mm. Out of 31 cases of invasive cancer, EIC was present around the invasive portion in 17 cases. The difference between the 'tumor size including EIC' and the 'invasive tumor size' indicates the extent of EIC spread (Table 2). Lesions number 33, 34, and 35, which are ILC, were accompanied by lobular carcinoma in situ (LCIS) as EIC.

**Table 1:** Patient and Tumor Characteristics

	N	%
Patients	32	
Mean age (range)	60.4 (34.8–87.1)	
Tumor foci	35*	
Invasive tumor size (mm)		
< 10	11	31
10–20	12	34
20 <	12	34
Tumor size, including EIC (mm)		
< 10	4	11
10–20	12	34
20 <	19	54
Histologic type		
Ductal carcinoma in situ	4	11
Microinvasive carcinoma	2	6
Invasive ductal carcinoma	25	71
Lobular carcinoma	4	11
Nuclear grade		
1	26	74
2	7	20
3	2	17
Intrinsic subtype		
Luminal A**	20	57
Luminal B	10	29
Her 2	1	3
Triple-negative	4	11

^*^One case had bilateral breast cancer and accessory lesions were present in two breasts

^**^The distinction between Luminal A and B is determined by the degree of malignancy, which is judged by factors such as the intensity of atypia and the value of Ki-67. There is a tendency for those with a Ki-67 value exceeding 20% to be assessed as Luminal B [6]

**Table 2:** Tumor Size on Each PET Threshold

Lesion no	Histology	Nuclear grade	Intrinsic subtype	Invasive tumor size (mm)	Tumor size including EIC (mm)	SUVmax	PET size (mm)			
							SUVmean + 2 SD	SUVmean + 3 SD	1.5 SUVmean + 2 SD	1.5 SUVmean + 3 SD
1	DCIS	1	Luminal B	0	4	NA	NA	NA	NA	NA
2	DCIS	1	Luminal A	0	11	2.9	23	20	15	15
3	DCIS	1	Luminal A	0	13	11.3	33	25	22	22
4	DCIS	1	Luminal A	0	110	4.6	110	107	96	92
5	Microinvasive carcinoma	1	Luminal A	1	14	10.7	24	21	17	16
6	Microinvasive carcinoma	1	Luminal A	1	25	11.2	20	20	19	16
7	IDC	1	Luminal A	5	6	7.3	11	9	7	4
8	IDC	2	Luminal A	6	6	2.7	13	9	7	6
9	IDC	1	Luminal B	8	8	2.5	12	12	9	7
10	IDC	1	Luminal B	12	12	9.1	20	17	16	16
11	IDC	1	Luminal A	12	12	5.4	26	20	18	16
12	IDC	2	Luminal A	13	13	5.1	27	21	19	17
13	IDC	2	Luminal A	15	15	8.5	16	16	16	14
14	IDC	1	TN	15	15	10.5	21	20	19	18
15	IDC	1	Luminal A	16	16	10.6	24	23	21	21
16	IDC	1	TN	15	20	7.3	34	31	20	17
17	IDC	1	Luminal B	21	23	8.6	32	32	29	29
18	IDC	1	Luminal A	10	24	7.4	40	29	22	21
19	IDC	3	TN	24	24	17.9	36	32	30	30
20	IDC	3	TN	14	25	17.8	25	21	20	20
21	IDC	1	Luminal A	25	25	11.7	29	29	27	24
22	IDC	1	Luminal B	24	26	5.3	30	27	23	18
23	IDC	1	Luminal A	30	30	2.8	43	29	29	24
24	IDC	1	Luminal B	34	34	8.6	29	26	26	26
25	IDC	2	Her2	7	35	3.6	35	29	23	21
26	IDC	2	Luminal A	12	37	3.1	23	19	11	10
27	IDC	1	Luminal A	30	38	20.2	31	32	31	30
28	IDC	2	Luminal B	22	40	12.2	35	34	32	32
29	IDC	1	Luminal B	42	42	9.2	49	49	48	41
30	IDC	1	Luminal A	23	45	11.8	42	42	40	40
31	IDC	1	Luminal A	34	46	10.0	63	51	44	31
32	ILC	2	Luminal B	12	12	3.4	20	14	12	11
33	ILC	1	Luminal A	2	20	2.2	17	13	8	7
34	ILC	1	Luminal B	12	23	2.8	22	22	21	19
35	ILC	1	Luminal A	22	55	3.2	45	33	26	21

*NA* not available, *TN* Triple negative

### Detection of breast cancer lesions

Table 2 shows the tumor size based on each PET threshold. There was a single case of 4-mm DCIS with a nuclear grade of 1, classified as low grade, that went undetected by PET, resulting in a sensitivity rate of 97%.

### Determination of the threshold for PET measurements

The inter-rater reliability for measuring the SUVmean of normal breast tissue between the two readers was high, with an ICC (Intraclass Correlation Coefficient) of 0.965 (95% confidence interval: 0.929–0.983).　In a comparison of 13 premenopausal and 18 postmenopausal cases, the average SUVmean value for normal breast tissue was found to be significantly higher (p < 0.05) in the premenopausal group at 0.89, compared to 0.65 in the postmenopausal group, as indicated by a Mann–Whitney U-test. Table 3 presents the mean values and ranges for four distinct threshold groups. The threshold values increase sequentially in the order of 'SUVmean + 2 SD', 'SUVmean + 3 SD', '1.5 SUVmean + 2 SD', and '1.5 SUVmean + 3 SD'.

**Table 3:** The Mean Value and Range of the Four Groups of Thresholds

Threshold type	Mean	Range
SUVmean + 2 SD	1.103	0.565–1.843
SUVmean + 3 SD	1.273	0.661–2.240
1.5 SUVmean + 2 SD	1.454	0.752–2.471
1.5 SUVmean + 3 SD	1.655	0.848–2.764

Analysis of all 32 cases

**Fig. 3:**
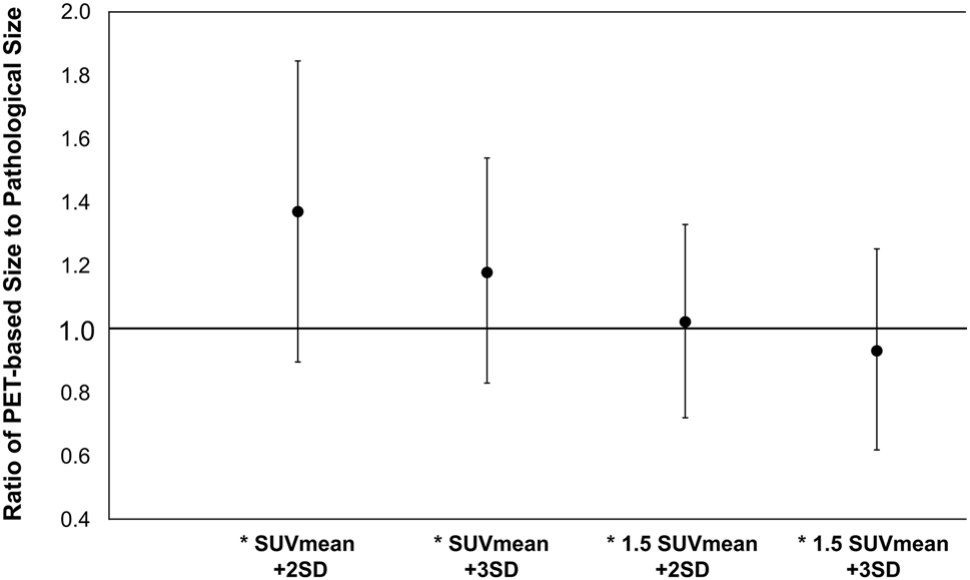
Rate of 'PET-based tumor sizes/pathological tumor sizes, including EIC' at each threshold. (Analysis of 34 lesions excluding Lesion no.1.). * Statistically significant differences were observed between all threshold groups (p < 0.05)

The measurement and analysis of tumor size was conducted on 34 cases, excluding Lesion no.1 which could not be detected. Given the considerable variation in pathological tumor sizes, ranging from 6 to 110 mm, we compared the ratio of the PET-based tumor size to the pathological tumor size (PET/Pathology) among the four threshold groups. Statistically significant differences were observed between all threshold groups (p < 0.05). Among the four groups, the group with the '1.5 SUVmean + 2 SD' threshold had the measured mean 'PET/Pathology' value closest to 1.0 (Fig. 3).

**Fig. 4:**
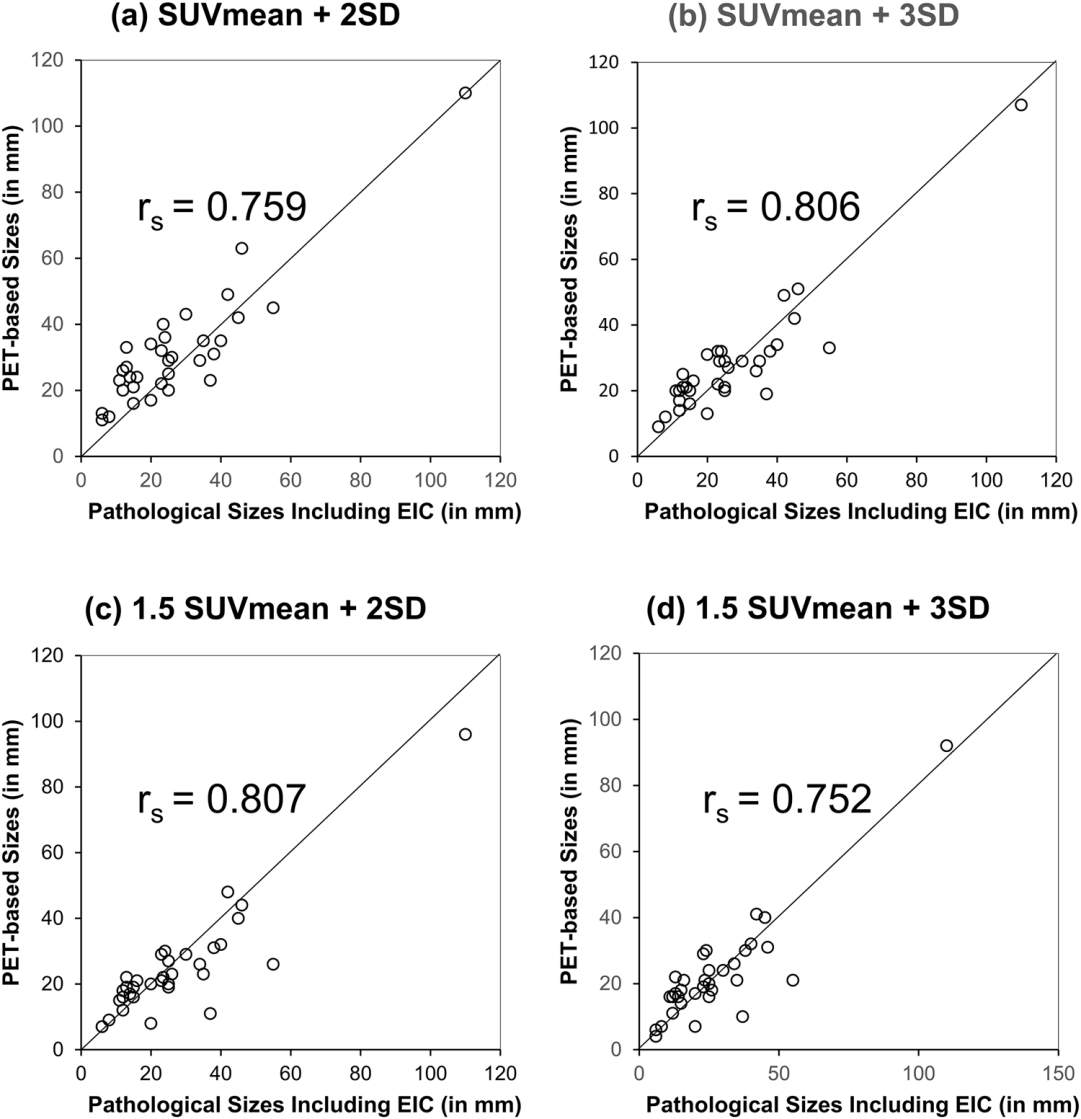
Diagram of the correlation between PET-based tumor size and pathological tumor size by Spearman’s rank correlation analysis. (Analysis of 34 lesions excluding Lesion no.1.). **a** Correlation between PET-based tumor size and pathological tumor size at the 'SUVmean + 2 SD' threshold. **b** Correlation between PET-based tumor size and pathological tumor size at the 'SUVmean + 3 SD' threshold. **c** Correlation between PET-based tumor size and pathological tumor size at the '1.5 SUVmean + 2 SD' threshold. **d** Correlation between PET-based tumor size and pathological tumor size at the '1.5 SUVmean + 3 SD' threshold. The '1.5 SUVmean + 2 SD' threshold shows the strongest positive correlation (r_s_ = 0.807) among the four thresholds

Figure 4 shows the correlation diagram between PET-based tumor size and pathological tumor size on each PET threshold by Spearman’s rank correlation analysis. The threshold of '1.5 SUVmean + 2 SD' shows the strongest positive correlation (r_s_ = 0.807) among the four thresholds. Moreover, the percentage of the size difference between PET and pathology (|1—PET/Pathology|) was compared among the four threshold groups (Table 4). Also, the threshold of '1.5 SUVmean + 2 SD' had the least deviation (23.1%) from pathology in the four groups.

**Table 4:** Deviation from Pathological Tumor Size at Each PET Threshold

Threshold type	Mean ratio (%) of	Range (%) of
	|1—PET/pathology |	|1—PET/ athology |
SUVmean + 2 SD	45.4	0–154
SUVmean + 3 SD	32.1	3–92
1.5 SUVmean + 2 SD	23.1	0–70
1.5 SUVmean + 3 SD	26.7	0–73

Analysis of 34 lesions excluding Lesion no.1

According to Table 5, measurements were considered concordant when they fell within ± 25% of the pathological tumor size, including EIC. In breast lumpectomy, a resection margin of about 2 cm is often taken. Therefore, we believe that the measurement error of tumor size should be kept below 25%. Among the four types of patient-specific threshold candidates, the concordance of PET-based tumor sizes and pathological tumor sizes was the highest (63%) with the threshold of '1.5 SUVmean + 2 SD'. At the thresholds of 'SUVmean + 2 SD' and 'SUVmean + 3 SD', the rate of overestimation increased. Conversely, at the '1.5 SUVmean + 3 SD' threshold, the rate of underestimation increased.

**Table 5:** Concordance (± 25%) between PET-based Tumor Sizes and Pathological Tumor Sizes at Each PET Threshold

	SUVmean + 2 SD	SUVmean + 3 SD	1.5 SUVmean + 2 SD	1.5 SUVmean + 3 SD
Underestimated	1 (3)	3 (9)	4 (11)	8 (23)
Concordant	15 (43)	17 (49)	22 (63)	19 (54)
Overestimated	18 (51)	14 (40)	8 (23)	7(20)
Not measurable	1 (3)	1 (3)	1 (3)	1 (3)
Total	35 (100)	35 (100)	35 (100)	35 (100)

The numbers in parentheses indicate percentages. Analysis of all 35 lesions

**Fig. 5:**
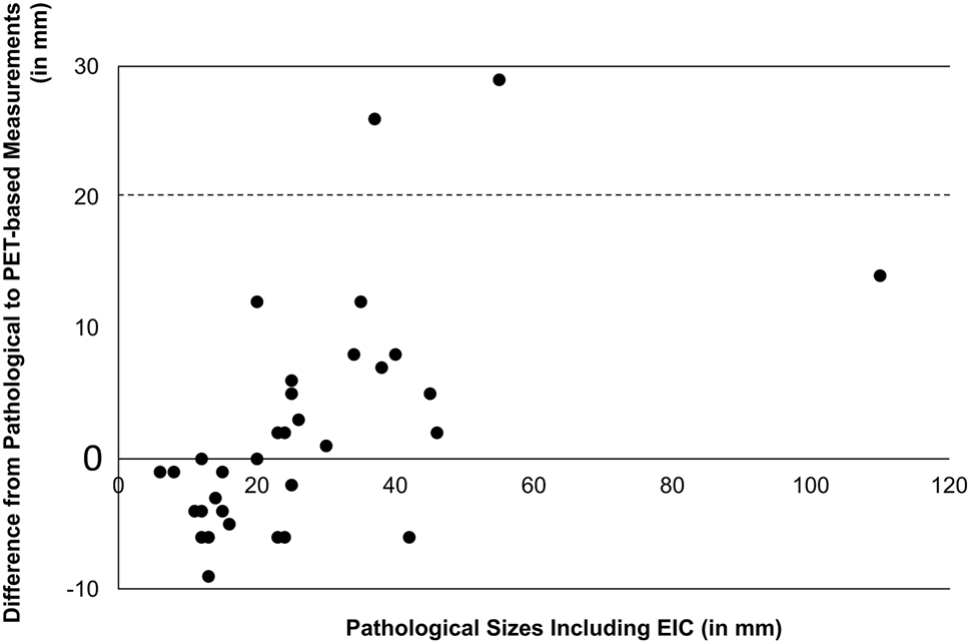
Difference from the pathological tumor size to the PET-based tumor size at the threshold of '1.5 SUVmean + 2 SD'. (Analysis of 34 lesions excluding Lesion no.1.). In 2 out of 34 lesions (Lesion 26 and Lesion 35), the difference between the pathological tumor size and the PET-based tumor size exceeded the standard resection margin of 20 mm for breast cancer partial mastectomy

Figure 5 shows the actual measurements of the difference from the pathological tumor size to the PET-based tumor size at the threshold of '1.5 SUVmean + 2 SD'. In 2 out of 34 lesions (Lesion 26 and Lesion 35), the difference between the pathological tumor size and the PET-based tumor size exceeded the standard resection margin of 20 mm for breast cancer partial mastectomy. Lesion 26 was histopathologically identified as IDC, while Lesion 35 was classified as ILC. In both cases, the extent of EIC exceeded 30 mm, and the intrinsic subtype was identified as Luminal A.

**Fig. 6:**
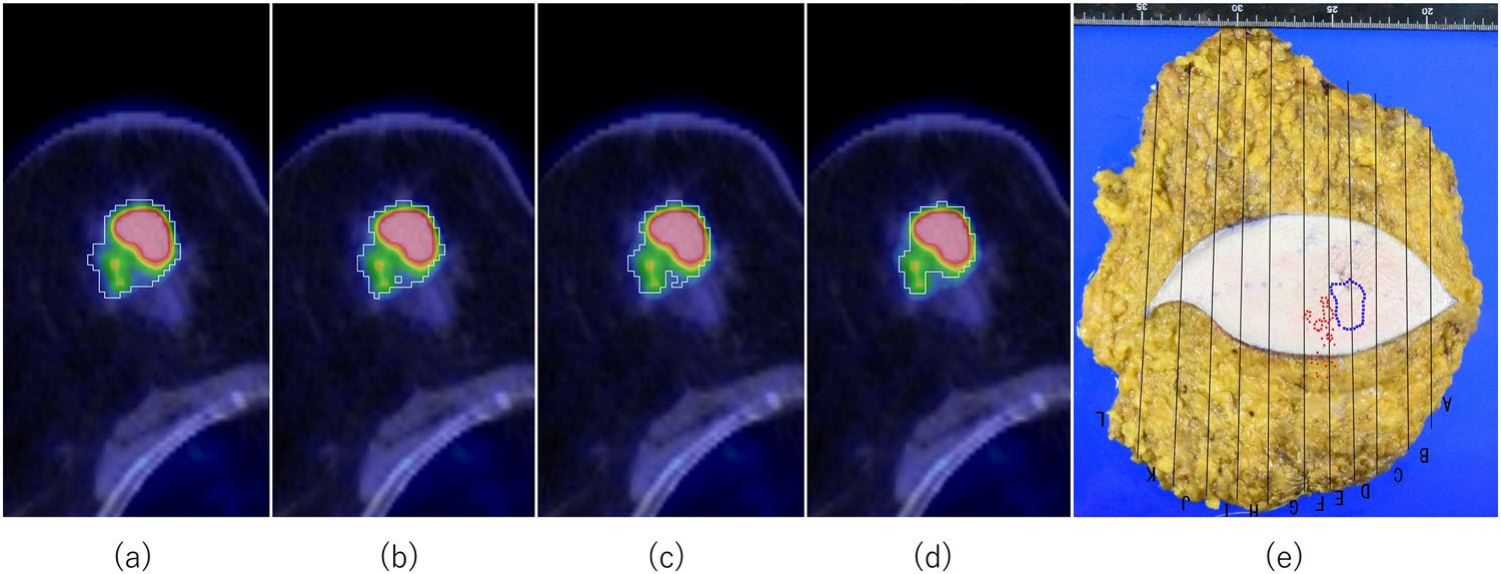
Semiautomatic delineation of tumor contours in Lesion no. 30. **a** 'SUVmean + 2 SD': threshold = 1.217, measured size = 42 mm. **b** 'SUVmean + 3 SD': threshold = 1.472, measured size = 42 mm. **c** '1.5 SUVmean + 2 SD': threshold = 1.57, measured size = 40 mm. **d** '1.5 SUVmean + 3 SD': threshold = 1.826, measured size = 40 mm. **e** Resected specimen (total mastectomy). The pathological diagnosis confirmed IDC. Including the EIC, the pathological tumor size was 45 mm. The blue dot represents the range of invasive carcinoma. The red dot represents the range of EIC. FDG uptake in background breast tissue was 'SUVmean = 0.707, SD = 0.255'

Representative images are shown in Fig. 6.

## Discussion

Among the four threshold groups, PET measurements at the '1.5 SUVmean + 2 SD' threshold demonstrated values closest to the pathological tumor sizes, including EIC. Also, the '1.5 SUVmean + 2 SD' threshold had the least deviation (23.1%) from pathology among the four groups. Moreover, a measurement was considered concordant if it fell within ± 25% of the pathological tumor size, and the threshold of '1.5 SUVmean + 2 SD' demonstrated the highest concordance rate of 63%.

Dynamic MRI, the most accurate breast imaging modality for assessing the extent of breast cancer, is useful for detecting ipsilateral and contralateral additional cancerous lesions; however, this detection could be rendered not feasible by contrast agent allergy, impaired renal function, implanted medical devices, or metallic foreign bodies [2, 7]. PET, on the other hand, does not have the side effects that are a concern with dynamic MRI.

Breast PET, with its high spatial resolution, was put to practical use in the 1990s and has been used clinically. Compared with contrast-enhanced MRI, breast PET has comparable sensitivity and slightly higher specificity for detecting breast cancer [8]. Conventional whole-body PET/CT using a photomultiplier tube detector (PMT PET/CT) has lower sensitivity for detecting lesions measuring less than 1 cm compared to breast PET. The low spatial resolution of the detector and the respiratory movement caused by imaging in the supine position are the causes of the low detection sensitivity for small lesions [9]. The prone position imaging employed in this study suppresses respiratory movements and increases lesion detection sensitivity [10].

SiPM PET/CT, which was put into practical use in the 2010s, has a higher spatial resolution than PMT PET/CT [11]. We assume that it is possible to diagnose the spread of cancer within the breast by imaging in the prone position using WB SiPM PET/CT. Breast PET has the disadvantage of having a blind area in the mammary glands near the chest wall because the detector cannot fully cover the anterior chest wall [12, 13]. However, WB SiPM PET/CT does not have this blind area near the chest wall and has the advantage of being able to simultaneously evaluate uptake in the regional lymph nodes.

In breast PET, imaging is performed at a position closer to the lesion compared to WB SiPM PET to increase the spatial resolution. Theoretically, the spatial resolution deterioration of the detector due to annihilation photon acollinearity is proportional to the detector ring diameter (2 R), and the magnitude of this deterioration (in mm FWHM) is given by 0.0044 R [14]. The deterioration of spatial resolution due to acollinearity in breast PET with a diameter of 200 mm is 0.44 mm FWHM, while that in WB SiPM PET/CT with a diameter of 600 mm is 1.32 mm FWHM. These values are constant regardless of the detector’s performance. However, the actual spatial resolution measured using a phantom in WB SiPM PET/CT is only about 5 mm FWHM [11]. Technological advances in detectors continue to improve the performance of WB SiPM PET systems, and there seems to be ample room for improvement in the spatial resolution of future WB SiPM PET/CT.

We used the tumor delineation method used by PERCIST as a reference to measure the tumor size. Hirata et al. placed a 30-mm spherical volume of interest (VOI) in the normal liver, measured SUVmean and SD, determined a patient-specific threshold, and delineated the tumor contour. Semiautomated tumor volume delineation software can delineate the contour of the tumor according to a certain threshold, which enables a reproducible evaluation of the tumor size. They stated that using a value of 'SUVmean + 3 SD' as the threshold is optimal because it is consistent with visual evaluation [4]. On the other hand, Wahl et al. propose the use of '1.5 SULmean + 2 SD' of the normal liver as a threshold [5, 15]. These PERCIST methods based on normal liver uptake values were proposed for the purpose of evaluating the volumes of tumors that had metastasised throughout the body. Therefore, the threshold value is large and cannot be applied as it is for the purpose of measuring the tumor size of early-stage breast cancer. We measured the SUVmean and SD of normal mammary glands as reference threshold values. The diameter of the spherical VOI was 20 mm, which is sufficiently within the dimensions of the normal mammary gland.　Furthermore, in addition to the thresholds of 'SUVmean + 3 SD' and '1.5 SULmean + 2 SD' used in the PERCIST criteria, we considered 'SUVmean + 2 SD' as a slightly lower threshold and '1.5 SULmean + 3 SD' as a slightly higher threshold, thus examining a total of four different threshold candidates.

This study had some limitations. First, this retrospective study represents our initial experience with a limited number of cases. In particular, there were only four cases of small lesions less than 10 mm, and we would like to accumulate data by increasing the number of cases in the future. A comparison with other imaging modalities will be necessary, and we aim to consider this in our subsequent research. Second, there was no particular trend observed in the correlation between the pathological classification and the tumor size on PET due to the small number of cases. We plan to increase the number of cases and continue our investigation in the future. Third, this study is a single-centre study; so, we would like to conduct more multicenter studies in the future. Furthermore, although not included in the present study, FDG uptake is often low in mucinous carcinoma, and it is considered that FDG-PET evaluation is not suitable.

## Conclusion

The study demonstrated that among various PET thresholds, the '1.5 SUVmean + 2 SD' threshold exhibited the best performance. However, even with this threshold, the concordance rate was limited to only 63%. We believe that this is the basis for diagnosing the spread of cancer within the breast using WB SiPM PET.
